# miR-378b Promotes Differentiation of Keratinocytes through NKX3.1

**DOI:** 10.1371/journal.pone.0136049

**Published:** 2015-08-27

**Authors:** Xi-liang Wang, Tao Zhang, Jing Wang, Dian-bao Zhang, Feng Zhao, Xue-wen Lin, Zhe Wang, Ping Shi, Xi-ning Pang

**Affiliations:** 1 Key Laboratory of Cell Biology, Ministry of Public Health of China, Department of Stem Cells and Regenerative Medicine, China Medical University, Shenyang, 110122, China; 2 Department of Anus and Intestine Surgery, the First Affiliated Hospital, China Medical University, Shenyang, 110001, China; 3 Department of Blood Transfusion, Shengjing Hospital of China Medical University, Shenyang, 110004, China; 4 Department of General Practice, the First Affiliated Hospital, China Medical University, Shenyang, 110001, China; University of Barcelona, SPAIN

## Abstract

MicroRNA (miRNA) is a kind of short non-coding RNA, involved in various cellular processes. During keratinocyte differentiation, miRNAs act as important regulators. In this study, we demonstrated by microarray assay that the expression of miR-378b significantly increased during keratinocytes differentiation. Our findings showed that miR-378b could inhibit proliferation, migration and differentiation in keratinocytes. Luciferase reporter assays showed that miR-378b directly target NKX3.1. Silencing of NKX3.1 could coincide with the effects of miR-24 overexpression. In conclusion, our results demonstrate miR-378b promote keratinocytes differentiation by targeting NKX3.1. Manipulation of miR-378b may afford a new strategy to clinic treatment of skin injury and repair.

## Introduction

The skin is the largest organ in the human body, and it is located at the interfaces of the internal and external environments of the body. Therefore, it provides an important barrier protection for the body [1]. Skin consists of the epidermis and dermis. The epidermis keeps self-renewing its whole life; the stem cells in the basal layer are proliferating and differentiating continuously to replace the terminally differentiated cells in the outer layer, thus realizing the renewal of tissue structures [2]. The death and exfoliation of cells in the outer layer and the division of stem cells in the basal layer are kept balanced to a certain degree, which is also the basic requirement for maintaining the normal tissue structures and a stable intracellular environment. Therefore, the epidermis is of great significance in the research on the differentiation of keratinocytes.

MicroRNA (miRNA) is an important type of non-coding RNA that is approximately 21–25 bp long. It highly conserves in the evolutionary process and widely presents in several biological process [3–11]. There are 2–8 relatively constant nucleotides at the 5’-termini of mature miRNA known as “seed sequences”, interacting with specific base pairing of target mRNA. The affection to the interaction causes the degradation of target mRNA and/or inhibits the translation of target gene mRNA, as a post-transcriptional regulation on the expression of target genes [12, 13]. The miR-203, miR-24, miR-17 and miR-31 have been shown as important regulators involved in the proliferation, differentiation and migration of keratinocytes [14–20].

Human miR-378b is located on chromosome 3. It is a member of the miR-378 family and has the same seed sequence as miR-378 (miR-378a). Previous studies show that miR-378 is involved in the regulation of the cell differentiation process [21, 22]. However, the relationship between miR-378b and keratinocyte differentiation is still unknown.

In the present study, we found that miR-378b were up-regulated during differentiation process of keratinocytes, and promoted cell differentiation through NKX3.1. These investigations provide a new molecular mechanism of differentiation process of keratinocytes.

## Materials and Methods

### Ethics

The skin grafts discarded after phimosiectomy were collected from the Department of Urinary Surgery of the No. 202 Hospital. This study was approved by the Ethics Committee of the No. 202 Hospital. Written informed consent was obtained from all of the patients before their participation in accordance with the Declaration of Helsinki.

### Isolated culture and differentiation of keratinocytes

The skin grafts were washed with D-Hanks (Invitrogen) to remove blood cells, then the subcutaneous adipose cells were removed, and the treated grafts were sheared into 1×1 cm ~ 2×1 cm tissue blocks. These tissue blocks were digested with 2 mg/mL neutral proteinase at 4°C for 10–12 h and further digested at 37°C for 3 h, and thereafter the epidermis was separated. The epidermis was sheared into pieces, digested with 0.25% trypsin-EDTA (Invitrogen) at 37°C for 20 min, combined with DMEM/F12 (Invitrogen) containing 10% FBS to terminate digestion, and blown into a single cell suspension. The cell suspension was grinded and filtered with a 200-mesh sieve. Thereafter, the cell suspension was centrifuged at 1000 rpm for 10 min to collect cells. The supernatant was removed, and the cells were washed once with D-Hanks and then suspended with Epilife medium (Invitrogen) into the plastic culture flask (NUNC) and incubated at 37°C for 20 min. The culture solution was replaced, the suspended cells and impurities were removed, and the cells adhering to the bottom of the culture flask were keratinocytes. The keratinocytes were further cultured in a 37°C, 5% CO_2_ incubator. When the cell confluency reached 70%-80%, cells were digested with trypsin for culture passage. The second- or third-generation cells were induced to differentiate by adding 1.5 mM CaCl_2_ (Sigma) to the culture medium.

### miRNA microarray

The miRCURY LNA Array (v.18.0) was used for detection. The slides were scanned using the Axon GenePix 4000B microarray scanner. All of the above works were completed by KangChen Bio-tech Inc.

### Real-time quantitative PCR (qPCR)

The Superscript III First-Strand Synthesis System kit (Invitrogen) was used for the reverse transcription reaction. The first strand of cDNA was synthesized from purified RNA by reverse transcription. First, a 10 μL reaction system was prepared with 1 μg RNA, 1 μL RT-primer, 1 μL 10 mM dNTP and DEPC-treated water and then reacted at 65°C for 5 min. Second, a 10 μL reaction system containing 2 μL 10×RT buffer solution, 2 μL 0.1 M DTT, 4 μL 25 mM MgCl_2_, 1 μL RNaseOUT and 1 μL SuperScript III RT was added and mixed evenly to prepare a 20 μL reaction system. The 20 μL reaction system was reacted at 50°C for 50 min and at 85°C for 5 min. Finally, 1 μL RNase H was added to react at 37°C for 20 min and then the reverse transcription product was collected. The reverse transcription reaction for miRNA was performed using miRNA qRT-PCR SYBR Kit (TaKaRa Bio). With the obtained cDNA as a template and using SYBR Premix Ex Taq II (TaKaRa Bio), qPCR was performed on ABI7500 (Invitrogen). The reaction condition was as follows: melting at 95°C for 10 s, annealing at 95°C for 5 s, and extension at 60°C for 20 s, for a total of 45 cycles. Expression levels of both mRNA and miRNAs were compared using 2^-ΔΔCT^ relative quantification method. The primer sequences were list in Table 1.

**Table 1:** Primers for qPCR analysis.

Gene symbol	Primer sequence
CK1-F	TCATCAACTACCAGCGCAGG
CK1- R	ACCATAACCACCACCAAAGC
CK10-F	AGGAGGAGTGTCATCCCTAAG
CK10-R	AAGCTGCCTCCATAACTCCC
Involucrin-F	TCCTCCAGTCAATACCCATCAG
Involucrin-R	CAGCAGTCATGTGCTTTTCCT
Filaggrin-F	CAATCAGGCACTCATCACAC
Filaggrin-R	ACTGTTAGTGACCTGACTACC
NKX3.1-F	CAGTCCCTACTGAGTACTCTTTCTCTC
NKX3.1-R	CACAGTGAAATGTGTAATCCTTGC
GAPDH-F	ACCACAGTCCATGCCATCAC
GAPDH-R	TCCACCACCCTGTTGCTGTA
hsa-miR-23b-5p- F	GGTTCCTGGCATGCTGATTT
hsa-miR-23b-5p- R	GTCGGTGTCGTGGAGTCG
hsa-miR-9-3p- F	GGATAAAGCTAGATAACCGAAAGT
hsa-miR-9-3p- R	GTCGGTGTCGTGGAGTCG
hsa-miR-149-3p- F	AAAGGGACGGGGGCTGT
hsa-miR-149-3p- R	GTCGGTGTCGTGGAGTCG
hsa-miR-4431- F	GCGACTCTGAAAACTAGAAGGT
hsa-miR-4431- R	GTCGGTGTCGTGGAGTCG
hsa-miR-378b- F	GGTGGACTTGGAGGCAGAA
hsa-miR-378b- R	GTCGGTGTCGTGGAGTCG
hsa-miR-595-F	GAAGTGTGCCGTGGTGTGTC
hsa-miR-595- R	GTCGGTGTCGTGGAGTCG
U6-F	GCTTCGGCAGCACATATACTAAAAT
U6-R	CGCTTCACGAATTTGCGTGTCAT

### Western blotting

Total protein was extracted using RIPA lysis buffer and then resolved by 10% SDS/PAGE followed by transferred onto polyvinylidene difluoride membranes (Hybond; GE Healthcare). The membranes were blocked in 5% (w/v) non-fat dried milk in PBST for 1 h and then incubated with primary antibodies and HRP-conjugated secondary antibodies. The following antibody dilutions were used: rabbit anti-GAPDH, (1:5000, Abcam), mouse anti-CK1 (1:1000, OriGene), mouse anti-CK10 (1:1000, Abcam), mouse anti-involucrin (1:2000, Abcam), rabbit anti-NKX3.1 (1:1000, Abcam).

### Bioinformatics

MicroRNA.org (http://www.microrna.org/microrna/home.do) and TargetScan 6.2 (http://www.targetscan.org) were used to determine binding sites in the 3’-UTR of NKX3.1.

### Luciferase assay and constructs

Two fragments (344–528, 911–1095) from the human NKX3.1 3’-UTR was synthesized by Sangon Biotech. Each fragment was subjected to restriction by XbaI and then cloned into the pGL3 control vector (Promega) to produce a wild-type reporter, termed pGL3-NKX3.1–1 and pGL3-NKX3.1–2, respectively. The mutant vectors, which were termed pGL3-mutNKX3.1–1 and pGL3-mutNKX3.1–2, were generated using the Site-directed Gene Mutagenesis Kit (Beyotime Institute of Biotechnology) according to the manufacturer's instructions. Primers for mutant site 1: forward: 5’-CCAACTGAATTAAACTTATCAGGTGAAGCCTCCTGT TGGCCT-3’ and reverse: 5’-AGGCCAACAGGAGGCTTCACCTGATAAGTTTA TTCAGTTGG-3’; Primers for mutant site 1: forward: 5’-ACTAAAGGGCTTCA TTTTCAGGTGATTTTTAGTCTGGCTGC-3’ and reverse: 5’-GCACCAGACTAAA ATCACCTGAAAAATGAAGCCCTTTAGT-3’. COS7 cells were spread evenly onto a 24-well plate. When these cells reached 60–70% confluence, 0.5 μg expression vectors containing the wild-type or mutant target sequences, 0.08 μg phRL-TK vectors, and 20 pM (final concentration) miR-378b mimic or NC were transfected into the cells within the experimental groups and control group using RNAiMAX according to the manufacturer’s protocol (Invitrogen). Luciferase activities of cellular lysis were measured 24 h after transfection by using a Dual-Luciferase Reporter Assay System (Promega). Light emission was measured using a luminometer.

### Oligonucleotide transfection

Keratinocytes were cultured to 60–70% growth, and then the culture solution was replaced. The miRNA mimics were synthesized by GenePharma. A short interfering RNA (siRNA) for NKX3.1 was obtained from Santa Cruz Biotechnology. Thereafter, miRNA mimic or siRNA was transfected into the cells according to the instructions using RNAiMAX.

### Construction and Transfection of NKX3.1 expression vector

The coding sequence of NKX3.1 cDNA was synthesized and inserted into the expression vector pcDNA 3.1 (Genecreate Biologlcal Engineering CO., China). Cells were transfected with NKX3.1 expression vector using lipofectamine 3000 according to the manufacturer's protocol (Invitrogen).

### Cell viability assays

Cell suspension (100 μl) was added into each well on a 96-well plate so that the density of tested cells was 3000/well. The 96-well plate was incubated at 5% CO_2_ and 37°C. The next day after cell adherence, the miR-378b mimic and siNKX3.1 were transfected and 3 parallel wells were set up. The cells were incubated at 5% CO_2_, 37°C for 24–48 h and then observed under an inverted microscope. MTT stock solution (20 μl of 5 mg/ml) was added into each well, and the culture was continued for 4 h. Thereafter, the culture was terminated, and the culture solution in the wells was carefully removed. Dimethyl sulfoxide (150 μl) was added into each well, and then the 96-well plate was placed on the shaker to swing at a low speed for 10min so that the crystals fully dissolved. The absorbance of each well was measured at OD 490 nm. All experiments were performed in triplicate and repeated three times.

### Wound scratch test

Cells were transfected and grown to confluency in 6-well plate. The next day, the scratches were made. The cells were washed with PBS three times to remove suspension cells and then cultured in a 37°C, 5% CO_2_ incubator. The cells were imaged at 0h, 12 h, 24 h, 48 h and 72 h.

### Statistics

All data were presented as means ± SD. Statistical analyses were performed using two-tailed Student’s *t* tests with SPSS 17.0 software.

## Results

### miR-378b expression was significantly increased in the differentiation process of keratinocytes

**Fig 1:**
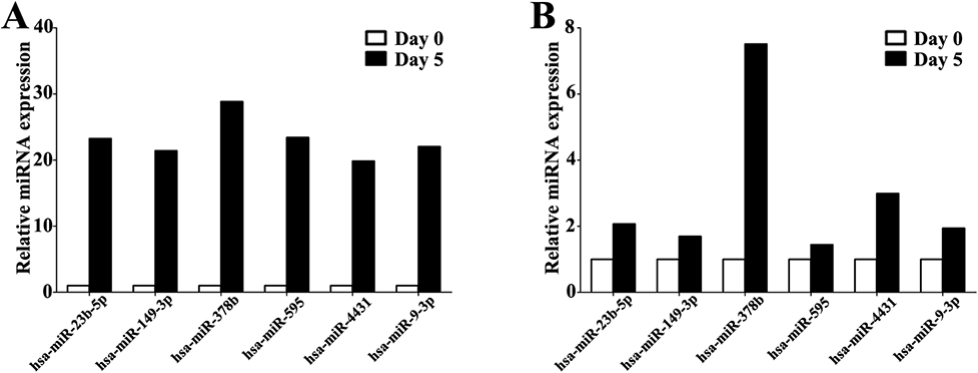
Comparison between microarray data and qPCR results. The expression levels of hsa-miR-23b-5p, hsa-miR-149-3p, hsa-miR-378b, hsa-miR-595, hsa-miR-4431 and hsa-miR-9-3p increased after calcium-induced for 5 days by microarray (A) were validated by qPCR (B). The data represent the means ± SD. All experiments were performed in triplicate. *P <0.05; ***P<0.001.

To determine the involvement of miRNAs in human epidermal differentiation, we exposed keratinocytes to high concentrations of calcium for 5 days to permit the development of terminal differentiation. Microarray analysis at various time points during differentiation revealed that several miRNAs were involved in the process. We found that the expression of 198 differentially expressed miRNAs were significantly up-regulated (Fold Change cut-off: 3.0), and the expression of 130 miRNAs were evidently down-regulated (Fold Change cut-off: 3.0). Six miRNAs that were significantly up-regulated were further validated by qPCR, which showed consistency with microarray data (Fig 1). According to the date, the expression of miR-378b increased most significantly. We thus hypothesized that induction of miR-378b is a critical event for epidermal development.

### miR-378b regulated the proliferation, migration and differentiation of keratinocytes

**Fig 2:**
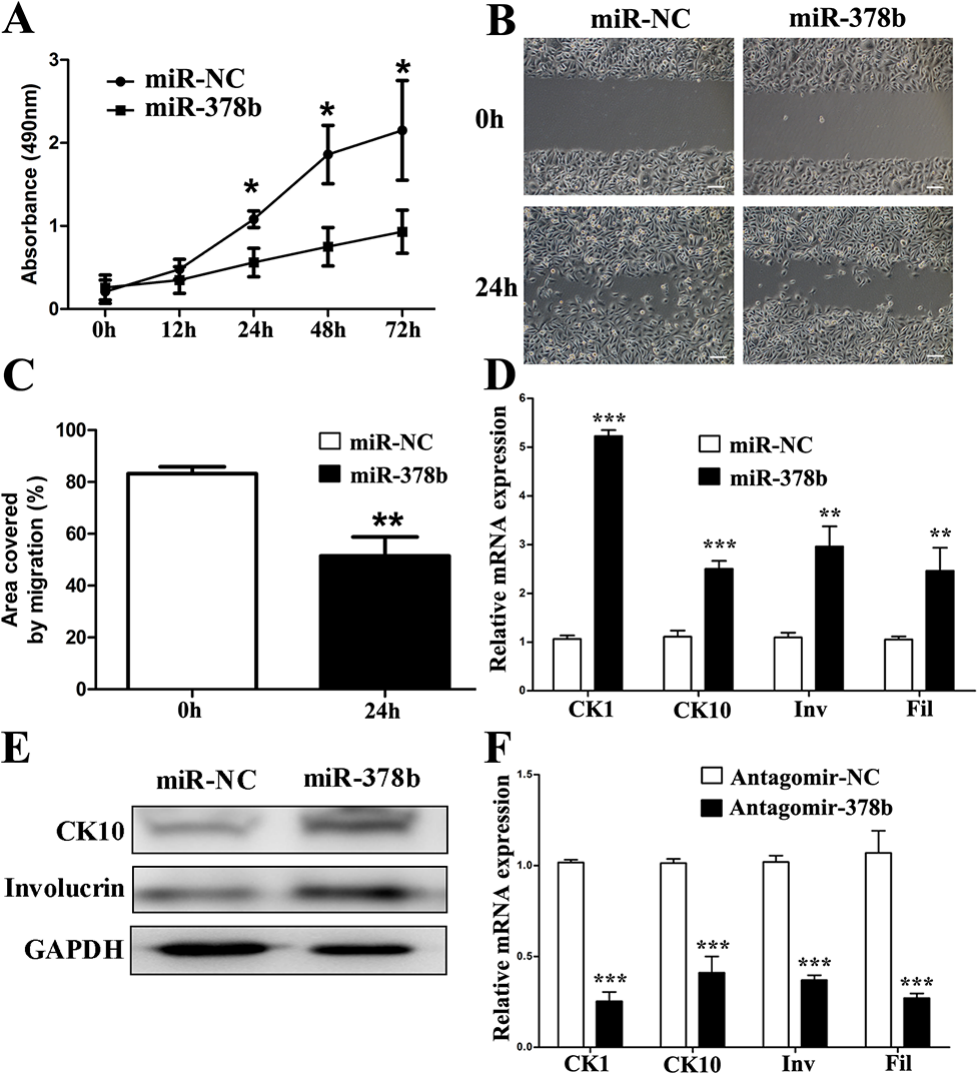
miR-378b regulates proliferation, migration and differentiation of keratinocytes. (A) Keratinocytes were transfected with miR-378b mimics, followed by cell viability assays at 0, 12 h, 24 h, 48 h and 72 h after transfection. (B and C) Forced expression of miR-378b caused a significant reduction in migration ability by the wound scratch assay, shown in the graph in C as area covered by migration during 24 h. Scale, 50 μm. CK1, CK10, involucrin (Inv) and filaggrin (Fil) expression levels were detected by qPCR (D) and western blot (E) after miR-378b over-expression for 5 days in proliferating conditions. (F) Real-time PCR analysis of CK1, CK10, involucrin and filaggrin mRNA levels after antagomiR-378b over-expression for 5 days in differentiating conditions. The data represent the means ± SD. All experiments were performed in triplicate. *P <0.05; **P<0.01; ***P<0.001.

To investigate the function of miR-378b in keratinocyte differentiation, we performed some experiments in primary human keratinocytes. As demonstrated by the MTT cell proliferation assay, the cell proliferation capacity of keratinocytes was decreased after miR-378b over-expression and further decreased to 50% of that in the control group at 72 h (Fig 2A). The wound scratch test confirmed a decrease in cell migration capacity after miR-378b over-expression (Fig 2B and 2C).

Over-expression of miR-378b promoted the differentiation of keratinocytes and increased the mRNA expression of keratin 1 (CK1), keratin 10 (CK10), involucrin and filaggrin (CK1: 5.2-fold; CK10: 2.5-fold; involucrin: 2.9-fold; filaggrin: 2.5-fold) (Fig 2D). That also increased the protein expression of CK10 and involucrin (Fig 2E). The miR-378b inhibitor antagonized the high calcium-induced expression of cell differentiation markers. Specifically, the mRNA expression of CK1, CK10, involucrin and filaggrin was decreased compared to the control group (CK1: 4-fold; CK10: 2.5-fold; involucrin: 2.7-fold; filaggrin: 2.7-fold) (Fig 2F). These results show that miR-378b promoted keratinocyte differentiation in vitro.

### NKX3.1 was a direct target of miR-378b

**Fig 3:**
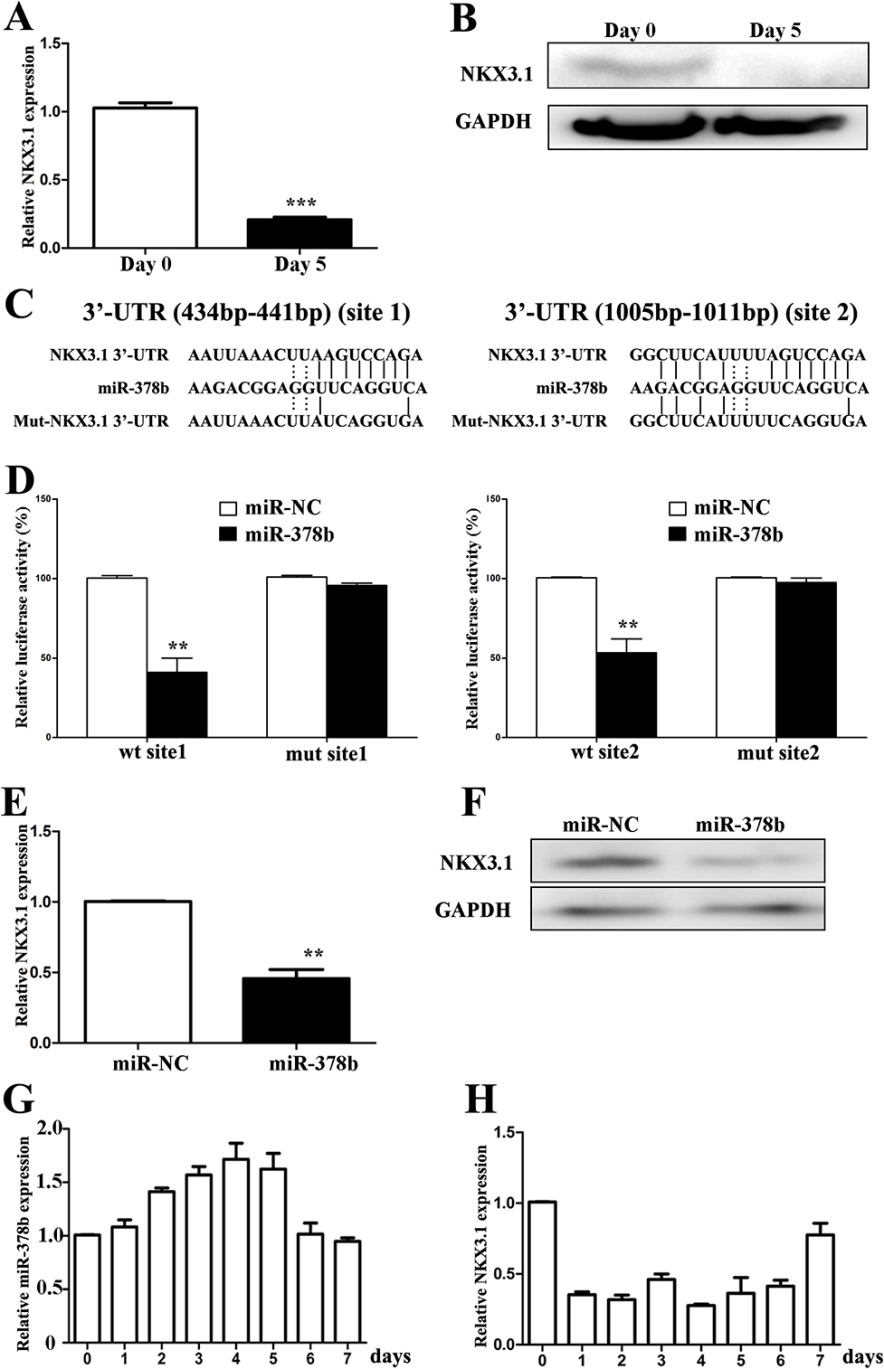
miR-378b directly targets NKX3.1. NKX3.1 expression levels were detected by qPCR (A) and western blot (B) for 5 days in differentiating conditions. (C) The alignment of miR-378b putative binding sites in human NKX3.1 3’-UTR region to show complementary pairing of miR-378b with NKX3.1 wild-type (WT) and mutant (Mut) 3’-UTR reporter constructs. The constructs were list blow: wt site 1: pGL3-NKX3.1–1; wt site 2: pGL3-NKX3.1–2; mutant site 1: pGL3-mutNKX3.1–1; mutant site 2: pGL3-mutNKX3.1–2. (D) COS7 cells were cotransfected with wt or mut reporter constructs and miR-378b mimics or miR-NC. The relative luciferase activities (ratios of firefly and renilla luciferase activity) of the lysates were measured by the dual luciferase reporter assay system (Promega, USA). Detection of NKX3.1 expression after miR-378b over-expression by qPCR (E) and western blot (F). Q-PCR showed miR-378b (G) and NKX3.1 (H) expression levels of keratinocytes for 7 days in differentiating conditions. The data represent the means ± SD. All experiments were performed in triplicate. **P<0.01; ***P<0.001.

To determine the miR-378b direct target gene, we screened NKX3.1 as a candidate by TargetScan and miRanda cross-comparison analysis. We observed that NKX3.1 mRNA expression was decreased by 80% after differentiation (Fig 3A). Western blot results also demonstrated a decrease in NKX3.1 protein expression after differentiation (Fig 3B).

There were two putative binding sites in the NKX3.1 3’-UTR region predicted by the software. To further examine whether miR-378b directly targets NKX3.1, we synthesized the two putative binding sites at positions 434–441 and 1005–1011, and six nucleotide substitutes in their seed regions (Mut) as indicated in Fig 3C, then cloned into the pGL3-control vector. For luciferase reporter assays, we co-transfected COS7 cells with reporter vector, phRL-TK vector and miR-378b mimic or negative control. The results suggested that miR-378b significantly suppressed the relative luciferase activity of both NKX3.1 wild type, but not mutant reporter activities (Fig 3D). These results indicated that miR-378b repressed NKX3.1 through two binding sites in the 3’-UTR region. Additionally five days after overexpression of the miR-378b, the mRNA and protein expression level of NKX3.1 was reduced (Fig 3E and 3F). To investigete the timescale for the increase in miR-378b and decrease in NKX3.1 during differentiation, a time point assay using qPCR for miR-378b and NKX3.1 was carried out 7 days in differentiating conditions every 24 hours. The results indicated that the expression of miR-378b was up-regulated during calcium-induced differentiation, though that was reduced on the sixth day (Fig 3G). Compatible with levels of miR-378b, the expression of NKX3.1 decreased at the beginning of differentiation, but increased on the fifth day (Fig 3H).

### miR-378b regulated the proliferation, migration and differentiation of keratinocytes through NKX3.1

**Fig 4:**
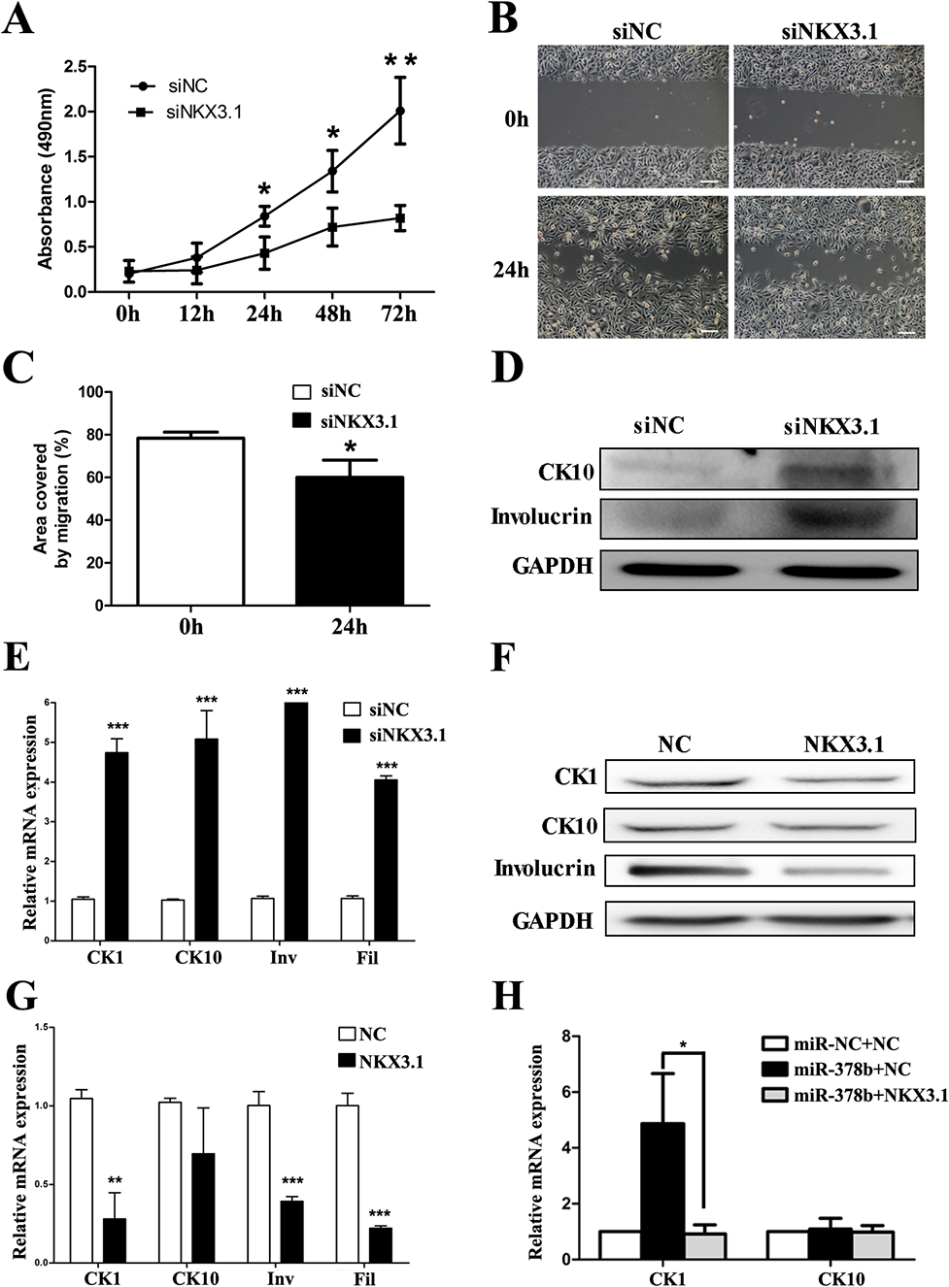
miR-378b regulates the proliferation, migration and differentiation of keratinocytes by targeting NKX3.1. (A) Keratinocytes were transfected with siNKX3.1, followed by cell viability assays at 0h, 12 h, 24 h, 48 h and 72 h after transfection. (B and C) Silencing of NKX3.1 caused a significant reduction in migration ability by the wound scratch assay, shown in the graph in C as area covered by migration during 24 h. Scale, 50 μm. CK1, CK10, involucrin (Inv) and filaggrin (Fil) expression levels were measured by western blot (D) and qPCR (E) after knock-down of NKX3.1 for 5 days in proliferating conditions. The differentiation markers were evaluated by western blot (F) and qPCR (G) after overexpression of NKX3.1 for 5 days in differentiating conditions. (H) The NKX3.1 expression vector was introduced into keratinocytes transfected with miR-378b mimics. NKX3.1 abolished miR-24 induced differentiation as shown by qPCR. The data represent the means ± SD. All experiments were performed in triplicate. *P <0.05; **P<0.01; ***P<0.001.

To investigate whether miR-378b promotes differentiation of keratinocytes by targeting NKX3.1, we performed NKX3.1 loss-of-function experiments. As demonstrated by the MTT cell proliferation assay (Fig 4A) and wound scratch test (Fig 4B and 4C), silencing of NKX3.1 showed a similar effect to miR-378b over-expression. The differentiation markers were also increased in proliferating condition (Fig 4D and 4E). Overexpression of NKX3.1 could reduce the expression of differentiation markers in differentiating conditions (Fig 4F and 4G), and repressed the effects of miR-378b on expression of CK1 (Fig 4H). These results show that miR-378b promote epidermal differentiation by targeting NKX3.1 in keratinocytes.

## Discussion

To identify miRNAs that regulate differentiation of keratinocytes, we performed a comprehensive miRNA expression profiling analysis before and after high calcium-induced differentiation of keratinocytes, we screened miR-378b which might participate in the regulation of differentiation. miR-378b is a member of the miR-378 family that is widely expressed in many tissues and it is particularly highly expressed in adipose tissues, heart and skeletal muscles. Jin W et al. also confirm the involvement of miR-378 in the regulation of adipogenesis [23]. So far, the relationship between miR-378b and keratinocyte differentiation has not yet been reported. Our experiment confirmed that the exogenous over-expression of miR-378b can promote the expression of the differentiation markers CK1, CK10, involucrin and filaggrin. Also miR-378b inhibitors could antagonize the high calcium-induced expression of cell differentiation markers. These results lead us to present that miR-378b is a critical regulator of keratinocyte differentiation.

NKX3.1 is a homeobox gene specifically expressed by prostatic cells. It is regulated by androgen and plays an important role in the prostate embryonic development [24], epithelial differentiation [25], and tumor occurrence [26]. Firstly our investigation indicated that NKX3.1 was expressing in keratinocytes, and its expression was reduced after differentiation. Secondly we demonstrated a novel link between miR-378b up-regulation and NKX3.1 suppression in keratinocytes. We found that miR-378b could inhibit NKX3.1 protein expressions through binding to 3’-UTR regions. Over-expression of miR-378b was sufficient to inhibit NKX3.1 protein levels.

NKX3.1 is a transcriptional repressor, and it inhibits cell proliferation in epithelial tissues of the prostate. It can inhibit the invasion and proliferation of the prostate cancer cell line PC-3 [27]. According to our experimental results, the regulation of NKX3.1 in keratinocytes is different. By cell proliferation and wound scratch assays, we found a decrease in the cell migration and proliferation capacities after knockdown by siNKX3.1 similar to the effects of miR-378b over-expression on keratinocytes. This may be related to different downstream genes regulated by NKX3.1. Our subsequent study will aim to thoroughly investigate other downstream genes regulated by NKX3.1 with the purpose of clarifying the specific mechanism for epidermal differentiation.

Keratin 1 is a member of the keratin gene family. It acts as early differentiation markers [19]. NKX3.1 recognizes consensus sequence (TAATTA)[28], and inhibits gene expression by directly binding to that site of the target genes. Through sequence alignment using “Patch” on the website (http://www.gene-regulation.com), we found that motif is located at -430bp to -425bp of NKX3.1 promoter. Combining with the results of known-down and overexpression, we assume that NKX3.1 may be as transcriptional repressor targeting CK1. Our subsequent study will investigate the relationship between CK1 and NKX3.1.

In addition, miR-378 over-expression inhibits osteoblast differentiation by targeted GALNT-7 and thus participates in the regulation of osteogenic differentiation [29]. With a dual-luciferase reporter system and RT-PCR, human cytochrome P450 family 2, subfamily E, polypeptide 1 (CYP2E1) is regulated by a miR-378 post-transcriptional mechanism [30]. By qPCR assay (data not shown), polypeptide N-acetylgalactosaminyltransferase 7 (GALNT-7) expression was decreased by 3.3-fold and CYP2E1 expression was increased by 2.5-fold after differentiation, indicating the possible involvement of GALNT-7 and CYP2E1 in the differentiation of keratinocytes.

In conclusion, our study has shown that miR-378b promotes the differentiation of keratinocytes by inhibiting NKX3.1 expression, and the repression of miR-378b expression can antagonize calcium-induced differentiation process. Therefore, miR-378b and NKX3.1 both appear to be important regulator in the epidermal differentiation process. The systematic study of miR-378b and NKX3.1 will be helpful to reveal the regulation mechanisms of epithelization in the skin injury and repair process, and drive future application of miRNAs in the clinical treatment of trauma.
